# Want doctors to use VR simulation? Make it mandatory, accessible, educationally valuable, and enjoyable!

**DOI:** 10.12688/mep.20040.2

**Published:** 2024-06-17

**Authors:** Riki Houlden, Fiona Crichton

**Affiliations:** 1 University of Dundee, Dundee, Scotland, UK; 2 East and North Hertfordshire NHS Trust, Stevenage, England, UK

**Keywords:** simulation, virtual reality, postgraduate doctor, foundation doctor, junior doctor

## Abstract

**Background:**

Virtual reality (VR) simulation training is mandatory for postgraduate year 1–2 doctors at the author’s hospital trust. Despite this, a preceding quantitative study demonstrated uptake below required levels. While the educational value of VR simulation has been highlighted, little attention has been paid to participant utilisation in postgraduate curricula. With the increasing development and incorporation of VR-based clinical education, it is essential to understand the factors influencing how frequently postgraduate doctors utilise it so that its potential can be maximised.

**Methods:**

A qualitative study design was employed. All 108 postgraduate year 1–2 doctors from the 2020-21 training year were invited for a semi-structured interview. Interviews continued until data saturation was reached in the form of informational redundancy. Reflexive thematic analysis was conducted.

**Results:**

A total of 17 interviews were conducted. Four main themes that influenced participation in VR simulation were identified: (1) the mandatory nature encouraged participation but led to negative perceptions as a tick-box exercise; (2) there were multiple challenges to accessing the resource; (3) the scenarios were felt to have limited educational value; and (4) there was untapped potential in drawing benefits from VR as an enjoyable leisure activity.

**Conclusions:**

Recommendations from these findings include: (1) VR simulation should be mandatory but with a degree of learner autonomy; (2) sessions should be integrated into doctors’ rotas as protected time; (3) more challenging scenarios ought to be created aligned with postgraduate courses, examinations, and specialty training, and (4) presented as a difficulty level system akin to gaming experiences.

## Introduction

Virtual reality simulation is growing. With technological advances and pressures on postgraduate training, the educational modality is increasingly being incorporated as a mandatory component of medical curricula. Despite this, there are to date no studies exploring its underutilisation by postgraduate learners.

Simulation-based medical education refers to any educational activity that utilises techniques or technology to mimic clinical scenarios. Virtual reality (VR) simulation is a subset of this and refers to the use of electronically generated visual environments to replicate real-world clinical scenarios ^1^. This can involve the use of a head-mounted display to achieve 3D immersion.

VR simulation training is a mandatory requirement for postgraduate year 1–2 doctors at the author’s hospital trust. The integration of VR training into the curriculum began in August 2019 and was part of a broader move in healthcare education towards VR simulation. The COVID-19 pandemic generated further interest nationally and internationally in its utilisation to minimise patient, student, and staff exposure to COVID-19 and to address the reduction in surgical training opportunities ^2, 3^. Associated with its incorporation are costs to hospital trusts, medical schools, and governing educational bodies for the head-mounted displays, laptops, and software licensing. Despite this, a preceding quantitative study ^4^ demonstrated that 5.7 scenarios were being completed per PGY1-2 doctor per year rather than the mandatory 10.

The educational value of VR simulation has been demonstrated in the literature ^5– 7^, leading to its incorporation in postgraduate endoscopic, surgical, and acute medical contexts ^5, 8^. However, little attention has been paid to participant utilisation of VR simulation since its introduction into postgraduate curricula. With the increasing development and incorporation of VR-based clinical education, it is essential to understand the factors influencing how frequently postgraduate doctors utilise this technology to maximise its cost-effectiveness.

### Problem statement

Postgraduate year 1–2 doctors do not make use of virtual reality simulation training as much as intended by their hospital trust.

### Research question

What factors influence how frequently postgraduate year 1–2 doctors make use of virtual reality simulation training?

## Methods

### Ethics

This study received ethical approval via the Integrated Research Application System on 4 ^th^ April 2022 (Project ID 311127). The research adhered to the tenets of the Declaration of Helsinki.

### Study design and rationale

The “What…?” question structure implies multiple truths that will be identified through the varying viewpoints of the participants, known as subjectivist epistemology ^9, 10^. This aligns with an interpretivist theoretical perspective which aims to gather a detailed account of a phenomenon through the multiple perspectives of those who have experienced it ^11^. Individual semi-structured interviews were conducted to explore these perspectives, with the interview guide (Appendix A, found as *Extended data* ^12^) founded on a five-step framework recommended by Kallio *et al.* ^13^.

### Setting

The study took place at a single NHS Trust in the East of England Foundation School, where foundation doctors (PGY1-2s) were mandated to complete 10 VR simulation scenarios each year for Annual Review of Competence Progression.

The scenarios were developed by Oxford Medical Simulation and involve running software on a desktop or a laptop. These scenarios are intended to be completed with a head-mounted display for 3D audio-visual immersion and for movement within the scenario by means of head motion detection, as well as a handheld clicker for selecting actions. Completion is also possible without this equipment using a mouse.

### Participation and data collection

All 108 PGY1-2s from the 2020–21 training year were sent an invite by email (Appendix B, found as *Extended data* ^12^). All who expressed interest were sent a participant information sheet (Appendix C, *Extended data* ^12^) and consent form (Appendix D, *Extended data* ^12^) to be signed before participation. Data collection began on 15 ^th^ April 2022. Interviews continued until data saturation was achieved in the form of informational redundancy. Interviews were audio-recorded, transcribed and de-identified on Otter.ai.

### Data analysis

Interview transcripts were analysed through reflexive thematic analysis on Microsoft Word (version 2311). The six-step structure provided by Braun and Clarke ^14^ served as the template to ensure quality and rigor.

### Researcher background

The lead author is a former PGY1-2 and at the time was a colleague of the interviewees. Drawbacks of this position include participants assuming the researcher already knows what the participants know and familiarity between the participant and researcher influencing responses for fear of judgement ^15, 16^; the associated risks of social desirability bias and restrained criticism were mitigated by encouraging participants to provide criticism to help improve the VR training for the future. Furthermore, being a peer or a part of the study group was deemed beneficial overall through building rapport, providing the researcher with familiarity of the setting, and facilitating recruitment for interviews ^15– 17^. This position also inevitably influences the lens through which the author viewed the data during analysis, but this is accepted and embraced as a valuable resource in the context of a qualitative paradigm of research ^18^. This work was completed as part of the lead author’s Masters in Medical Education, under the supervision of the co-author.

## Results

There were 30 responses out of a possible 108 (28% response rate), with 28 of the 30 doctors volunteering to participate. A total of 17 interviews were conducted, terminating before 28, having satisfactorily achieved informational redundancy in the form of data saturation ^19^. Seven were PGY1s and ten were PGY2s. Four main themes emerged – VR simulation:

1. As a mandatory requirement – the mandatory nature encouraged participation but led to negative perceptions as a tick-box exercise
2. As an inaccessible resource – there were multiple barriers to access that needed to be overcome.
3. As an educational tool – participants had varying perceptions of the educational value of the resource, with many wanting scenarios of greater difficulty.
4. As a leisure activity – VR simulation was likened to a game, with untapped potential for a greater degree of competitive goal-orientation to be incorporated.

### A mandatory requirement

Most participants stated their only reason for doing VR simulation was because it was mandatory. The prevailing opinion was that this was appropriate: “if you don’t make it compulsory people won’t engage with it and actually, it’s a good resource to use” [Dr 4]. However, the associated removal of self-direction led to VR simulation being perceived negatively as a “tick-box exercise” [Dr 3]; participants wished to have a greater degree of autonomy to address their own learning needs. Solutions to reconcile this included: having “five [VR scenarios] that are compulsory and five you choose yourself” [Dr 11]; a couple of mandatory scenarios to facilitate initial engagement followed by “a bank of scenarios that you leave up to your learner to make decisions if they want to do them or not” [Dr 9]; or making the scenarios completely optional but “you can count them towards your [annual required] teaching hours” [Dr 12].

### An inaccessible resource

Booking for VR simulation sessions at the education centre was a common source of frustration, with participants needing “to organise with three or four different people to actually attend the VR sim” [Dr 15]. Some therefore appreciated having sessions booked for them automatically: “[They] just gave us a slot which was good in the sense that it didn’t rely on the disorganised [to book in]” [Dr 1]. Others disagreed: “I prefer booking in yourself. I think when you're allocated a date, sometimes it can be quite difficult to try and swap” [Dr 4]. Yet others suggested “a drop-in session would be quite useful” [Dr 11] as “having the availability to be flexible is one of the benefits of VR sim” [Dr 8]. Therefore, a mix of all three approaches may be necessary to maximise attendance.

Even after booking however, PGY1-2s were often unable to attend due to a clinical emergency, overwhelming workload, or understaffing. Accordingly, Dr 15 expressed that mandatory training “should just be timetabled in” and the corresponding “work shifts should just be, you know, cancelled”, thereby ensuring that alternative staffing cover has been arranged.

For the reasons above, many PGY1-2s found it considerably more convenient to complete scenarios at home in their own time. An initial barrier to this was incompatibility between the software and home devices: “there was no sort of way of to do them on a Mac laptop at home” but this was addressed by 2020–21 and “that made it a lot easier to engage with them” [Dr 2].

Following this step, some PGY1-2s struggled to navigate themselves within the VR learning environment, particularly when using a head-mounted display. Having “someone there that was able to help you” [Dr 5] at the education centre was valuable in overcoming this so home use would only be advisable after an initial session in-person. Alternatively, suggestions were made for a short “video tutorial” or a “sandbox mode” [Dr 10] in which learners could orient themselves to the environment and features available prior to their first scenario.

A barrier for several participants following orientation to scenarios was discomfort from wearing the head-mounted display. Needing to wear spectacles underneath was expressed to be a problem by Drs 2, 3, 7, and 12, and others described experiencing a mild headache (Dr 13) or even prohibitive motion-sickness (Drs 3 and 10). Advice must be provided to minimise the likelihood and severity of discomfort induced by the head-mounted display.

### An educational tool

Participants ranged widely from those who found the VR simulation scenarios to be “very interesting and educational” [Dr 9] to those who “[didn’t] find it very valuable” [Dr 17]. For example, features of the scenario that were already embedded as second nature such as handwashing and taking a history were felt to be of minimal educational value, particularly by PGY2s. There was instead demand for scenarios that supported PGY1-2s in their career development, through cases aligned with postgraduate courses (“that would be super cool to have some aspects of the [Advanced Trauma Life Support] course” [Dr 13]), examinations (“I thought it could be useful for ‘Part I’ revision” [Dr 6]), and specialty interests (“I think it would be great to have it tailored to a particular specialty” [Dr 15]). Interviewees highlighted that such scenarios might be damaging to confidence for some PGY1-2s so would need to be optional.

The head-mounted display facilitated engagement in the scenarios through providing audio-visual block-out from environmental distractions: “if you're just on the computer … you have people messaging you about patients and stuff on the ward or just other stuff and it's, you're not really taking in what you're actually doing” [Dr 11]; “it really kind of enables you to immerse yourself in the scenario” [Dr 4]. However, PGY1-2s felt there was no intrinsic educational value beyond this.

### A leisure activity

Many participants found VR simulation to be enjoyable, describing how it became “like a game” [Dr 4] when wearing the head-mounted display. Some interviewees described themselves as intrinsically competitive people that enjoyed this element in gaming, suggesting that this be incorporated into VR simulation through a “difficulty level” [Dr 7] system, whereby progressively more difficult (optional) scenarios would be unlocked on attaining a pre-determined pass score: “I'd love that, yeah, I think I'm very much a goal-oriented person so if you tell me I need to get to level 10 I will get to level 10” [Dr 14]. This concept was linked by interviewees to the previously discussed idea of creating more challenging scenarios aligned with postgraduate courses, examinations, and specialty training.

## Discussion

Although there is a broad literature base for the educational value of VR simulation, little work exists on the factors affecting its utilisation upon integration into postgraduate curricula. The main themes that arose were to make VR simulation mandatory, accessible, educationally valuable, and enjoyable, with learners providing recommendations on how these might be best achieved.

### A mandatory requirement

The mandatory requirement to complete 10 VR simulation scenarios acted as the greatest motivator for participants. This finding echoes Brooks and colleagues’ ^20^ study of PGY1-2 doctor perceptions of mandatory (non-VR) e-learning that had been integrated into their curriculum. Due to the impacts of COVID-19 however, this mandatory requirement for VR training was modified to being a non-mandatory expectation at our hospital trust. This led to many PGY1-2s completing no further scenarios. Going forward, clear mandates are recommended for successful implementation of VR training in the postgraduate curriculum ^8^.

Conflictingly, participants described how the mandatory (or strongly expected) nature of the VR simulation led to it being perceived negatively as a “tick-box exercise”, again observed by Brooks and colleagues ^20^. It is recognised that junior doctors perceive compulsory activities negatively when they are not felt to be relevant to one’s needs ^21, 22^ and a meta-analysis of VR training in nursing students found that when VR simulation was not self-directed it resulted in lesser improvements in knowledge ^7^. The theoretical underpinning for this lies in Knowles’ first of six assumptions underlying andragogy, which states that adults generally wish to choose what (as well as when and how) they learn ^23^. Therefore, while the mandates for VR training must be clear, they ought not to be restrictive – they should include a degree of obligation to ensure initial engagement, but with autonomy beyond this to enable postgraduate doctors to identify and address their own learning needs.

### An inaccessible resource

Despite PGY1-2s being expected to complete scenarios, the bookings system impeded attendance. The optimal booking system would be a mixed approach, again aligning with Knowles’ ^23^ first andragogical assumption that adults generally wish to choose when (as well as what and how) they learn. There will however be some doctors who are indifferent to this detail and would prefer to minimise their personal administrative burden, for whom automatic booking would be most appropriate.

A further challenge to attendance even after successful booking was leaving one’s clinical commitments. This problem has been recognised by other authors ^8, 20^, who suggested disciplinary action, a rewards system for compliance, and to provide PGY1-2s with protected time to attend during paid employment. As PGY1-2s struggled to leave clinical commitments due to emergencies, overwhelming workload, and understaffing, the first two of these suggestions are ethically problematic, encouraging doctors to prioritise personal interests over those of patients and colleagues. The third suggestion for protected time with alternative staffing cover arranged does not pose this moral dilemma; it was voiced by one of the interviewees in our study and is likely to be the most appropriate but would need the support of hospital and educational authorities.

To circumvent this, many PGY1-2s attempted to complete scenarios at home in their own time. An initial barrier to this was software incompatibility; technical problems are widely recognised in the VR literature ^6, 7, 24^ and are to be expected with new technologies. It is crucial that these are identified and addressed early to enable learner access to VR training.

Some PGY1-2s accessing the software at home struggled to navigate themselves within the VR learning environment without the support of staff at the education centre. A learner’s intention to use technology is influenced by its perceived ease of use ^25^ so efforts must be made to establish this perception. In addition to support staff for those completing scenarios in-person ^6^, a short “video tutorial” or a “sandbox mode” are recommended to support learners accessing VR alone from home.

Though many PGY1-2s preferred to wear the head-mounted display for its associated greater enjoyment and immersion, some found this mildly or even prohibitively uncomfortable. These effects are widely recognised and suggestions to minimise symptoms exist such as physical stabilisation of the learner in the real world and frequent breaks without the head-mounted display ^7, 24^. These recommendations must be incorporated into VR training programmes to maximise learner utilisation of head-mounted displays.

### An educational tool

Features of the scenario already embedded as second nature were felt to be of minimal value, which was noted to be more of an issue for PGY2s than PGY1s. This reflects the existing VR literature, with lower perceived and actual educational benefit in the training of learners at a more advanced stage of training than in novice learners ^26, 27^. Junior doctors often feel they receive inadequate support towards future training needs and postgraduate examinations ^22, 28^ and accordingly, there was demand from PGY2s for more challenging scenarios specifically designed to prepare for these.

Participants felt that the head-mounted display facilitated engagement in the scenarios through providing audio-visual block-out from environmental distractions, as supported by other VR studies comparing learning with and without head-mounted displays ^29^. Despite this, interviewees did not feel the head-mounted display held any intrinsic educational value. In contrast, Gutiérrez and colleagues ^30^ demonstrated that learners wearing a head-mounted display underwent a greater increase in knowledge than learners who were not, suggesting they may have intrinsic educational value. Their study did not ask learners for their perceptions however, so it is unclear whether they too felt that there was no educational benefit to the head-mounted display. If they had expressed the same opinion, this might indicate that there is an educational benefit of which learners are not consciously aware. Further research is required to clarify this; if a subconscious educational benefit is confirmed, this ought to be communicated to postgraduate trainees to improve learner buy-in ^25^.

### A leisure activity

One of the key features of VR simulation that made it attractive to learners was its enjoyable nature, as previously noted in the literature ^31^, likening it to gaming experiences. Participants described the competitive goal-oriented nature of games as one of the key elements that made them enjoyable and so suggested a difficulty level system, where progressively more challenging scenarios are unlocked based on performance. In addition to motivating postgraduate trainees through greater enjoyment, there appears to be educational value to this approach: when VR endoscopy cases were presented in order of increasing difficulty, this led to improved technical skills acquisition than when they were presented randomly ^32^.

### Strengths and limitations

Data were collected until data saturation was achieved in the form of informational redundancy. It is possible that some ideas were missed through nonresponse bias; this could have been addressed through repeated contact of all PGY1-2s but this was not pursued due to the risk of feelings of coercion. Reasons for non-participation may include being too busy with other commitments and not having an interest in being interviewed in the absence of perceived personal gain. Two PGY1-2s who did not wish to be interviewed as they had not completed any VR scenarios provided their perspectives via email and these were no different to the themes identified in the interviews.

This study investigates foundation year doctors at a single hospital trust participating in use of a single company’s VR simulation software. Our results are not generalisable to other training grades, medical students, or non-medical healthcare professionals, nor to other VR simulations with different intended learning outcomes.

Nevertheless, the results may be transferable to PGY1-2s at other hospital trusts and could inform changes in policy or practice at other institutions. Many of the above suggestions would be pre-emptively addressed and iteratively improved through following the BUILD REALITY framework ^33^ for the introduction of a VR-based medical simulation environment into curricula.

## Conclusions

The educational benefits of VR simulation are well-recognized, and our study uniquely explores its underutilisation following integration into postgraduate curricula. The main ways this can be addressed are to set clear mandates with a degree of learner autonomy and to make VR training more accessible, educationally valuable, and enjoyable. Hospital trusts, medical schools, and governing educational bodies that incorporate these themes, such as through following the BUILD REALITY framework, are likely to find their learners derive greater benefit from and more frequently utilise virtual reality simulation training.

## Data Availability

Figshare: Want doctors to use VR simulation? Make it mandatory, accessible, educationally valuable, and enjoyable! - Transcripts compiled. https://doi.org/10.6084/m9.figshare.24908505 ^19^. Figshare: Want doctors to use VR simulation? Make it mandatory, accessible, educationally valuable, and enjoyable! - Appendices.pdf. https://doi.org/10.6084/m9.figshare.24886992 ^12^. Data are available under the terms of the Creative Commons Zero "No rights reserved" data waiver (CC0 1.0 Public domain dedication).
