# Supplementary material for: Want doctors to use VR simulation? Make it mandatory, accessible, educationally valuable, and enjoyable!
Source: MedEdPublish (2016). 2024 Jun 17;14:8. Originally published 2024 Mar 1. [Version 2] doi: 10.12688/mep.20040.2 (PMC11200059; doi:10.12688/mep.20040.2)
Supplement: Peer review history

# Reviewer report: Reviewer response for version 2

**DOI:** 10.21956/mep.21885.r37564

**Published:** 2024-06-25

**Authors:** Kavin Selvan

**Affiliations:** 1 VCU School of Medicine, Virginia Commonwealth University, Richmond, Virginia, USA

Thanks for your comprehensive response, and for making the suggested revisions. It is more than suitable for Indexing.

Have any limitations of the research been acknowledged?

Yes

Is the work clearly and accurately presented and does it cite the current literature?

Partly

If applicable, is the statistical analysis and its interpretation appropriate?

Not applicable

Are all the source data underlying the results available to ensure full reproducibility?

Yes

Is the study design appropriate and is the work technically sound?

Partly

Are the conclusions drawn adequately supported by the results?

Yes

Are sufficient details of methods and analysis provided to allow replication by others?

Partly

Reviewer Expertise:

Qualitative, Quantitative, and Mixed Methods; VR in Medicine

I confirm that I have read this submission and believe that I have an appropriate level of expertise to confirm that it is of an acceptable scientific standard.

# Reviewer report: Reviewer response for version 1

**DOI:** 10.21956/mep.21457.r36854

**Published:** 2024-05-10

**Authors:** Kavin Selvan

**Affiliations:** 1 VCU School of Medicine, Virginia Commonwealth University, Richmond, Virginia, USA

This study explores the underutilization of mandatory virtual reality (VR) simulation training among postgraduate year 1-2 doctors at a single NHS Trust, identifying barriers to its effective use. The work is timely given the growing use of VR in medicine. The manuscript could be enhanced by considering the following adjustments. 

**Introduction:**

 The introduction effectively defines the use of VR simulation in medical education. However, the immediate definition of simulation-based medical education and VR simulation should not replace an engaging opening sentence. This could be strengthened by explicitly stating the research gap early on, particularly focusing on the specific issue of underutilization despite mandatory implementation. Further, the linkage between VR's potential benefits and its actual use in educational settings can be made more explicit to strengthen the study's rationale.

**Methods:**

***Ethics: *** The mention of ethical approval is appropriate; however, please specify which local IRB/REB granted approval and confirm that the research adhered to the tenets of the Declaration of Helsinki.

***Study Design and Rationale:*** The use of qualitative design is justified, yet the rationale could benefit from more depth regarding why this approach is specifically suitable for exploring the nuanced perceptions of medical education and/or VR utilization.

***Setting:*** The description is adequate.

**Participation and Data Collection:** The process is well-detailed.

***Data Analysis: *** Reflexive thematic analysis was employed following Braun and Clarke’s framework. To improve the reliability and validity of the findings, it would be helpful to specify any measures undertaken, such as double-coding of transcripts by multiple researchers or seeking external validation of the themes identified would strengthen the paper.

***Researcher Background: *** Acknowledging the researcher's positionality is good practice. However, the lead author's position as a former colleague of the interviewees might have influenced the responses, leading to social desirability bias or restrained criticism. The study acknowledges this but does not clearly detail how this potential bias was mitigated beyond recognizing it and mentioning its utility in developing rapport.

**Results:**

 The results are clearly structured around identified themes, which are relevant and significant to the study's aims. However, the response rate and reasons for non-participation could be included to assess potential bias. The study interviewed only 17 of the entire potential sample of 108 doctors. Although data saturation was reported to be achieved, the relatively small sample size and potential for non-response bias raise questions about the representativeness of the sample for the NHS Trust in the East of England Foundation School. This is particularly significant given the potential diversity of experiences and opinions within a larger cohort. Improvements could also be made by including a demographic table of the study participants.

**Discussion:**

 The recommendations made (e.g., making VR simulations both mandatory and non-restrictive, integrating them into doctors' schedules, and improving access to and relevance of scenarios) are logical extensions of the findings. The discussion effectively connects these findings to existing literature, offering insight of how the identified factors impact VR simulation use. Although the findings may not be generalizable due to the sample size, the discussion could be enhanced by including a broader consideration of implications for practice, particularly how these findings could inform changes in policy or practice at other institutions. This can also be addressed in the conclusion.

**Conclusions:**

 The conclusion is well-written. However, please provide more explicit clarification on how this study addresses the question of why VR simulations are underutilized in medical education (e.g., "The educational benefits of VR simulation are well-recognized, and our study uniquely explores its underutilization following integration into postgraduate curricula"). Furthermore, suggest a framework or study ^1^ for implementing VR simulation in the discussion, and reiterate it in the conclusion. Institutions could then adopt this framework to improve their VR utilization in medical education.

**References:**

 Reference 4: Houlden R. Please specify if this is a publication or conference, and include link.

 Reference 8: Illing J. Please include link, if possible.

 Reference 28: Dawson CR. Please include link, if possible.

Have any limitations of the research been acknowledged?

Yes

Is the work clearly and accurately presented and does it cite the current literature?

Partly

If applicable, is the statistical analysis and its interpretation appropriate?

Not applicable

Are all the source data underlying the results available to ensure full reproducibility?

Yes

Is the study design appropriate and is the work technically sound?

Partly

Are the conclusions drawn adequately supported by the results?

Yes

Are sufficient details of methods and analysis provided to allow replication by others?

Partly

Reviewer Expertise:

Qualitative, Quantitative, and Mixed Methods; VR in Medicine

I confirm that I have read this submission and believe that I have an appropriate level of expertise to confirm that it is of an acceptable scientific standard, however I have significant reservations, as outlined above.

# Reviewer report: Reviewer response for version 1

**DOI:** 10.21956/mep.21457.r36550

**Published:** 2024-05-07

**Authors:** Francis J Real

**Affiliations:** 1 Cincinnati Children's Hospital Medical Center, University of Cincinnati College of Medicine, Cincinnati, Ohio, USA

This qualitative study explores the factors impacting utilization of virtual reality (VR) curricular materials by post-graduate doctors. It succinctly and accurately describes a common challenge when implementing VR-based education among busy clinicians and provides pragmatic solutions. The authors provide a thorough description of their methods and analysis approach along with a supplementary interview guide. A table with exemplar quotes may help provide additional support for described themes to prevent readers from having to review full transcripts.

Have any limitations of the research been acknowledged?

Yes

Is the work clearly and accurately presented and does it cite the current literature?

Yes

If applicable, is the statistical analysis and its interpretation appropriate?

Not applicable

Are all the source data underlying the results available to ensure full reproducibility?

Yes

Is the study design appropriate and is the work technically sound?

Yes

Are the conclusions drawn adequately supported by the results?

Yes

Are sufficient details of methods and analysis provided to allow replication by others?

Yes

Reviewer Expertise:

Virtual reality simulation in post-graduate medical education

I confirm that I have read this submission and believe that I have an appropriate level of expertise to confirm that it is of an acceptable scientific standard.
